# Open spina bifida characterisation in a dog foetus

**DOI:** 10.1002/vms3.1266

**Published:** 2023-09-13

**Authors:** Yue Chen, Dan Su, Xiaorong Sun, Wenjuan Gui

**Affiliations:** ^1^ Chengdu Bio‐HT Company Limited Chengdu Sichuan China; ^2^ General Hospital of Ningxia Medical University Yinchuan Ningxia China

**Keywords:** congenital malformation, deformation of the frontal bone, hydrocephalus, meningomyelocele, open spina bifida

## Abstract

**Background:**

Open spina bifida is an uncommon malformation in animals, and there is a lack of imaging, clinical, and pathological characterisation of this condition in dogs.

**Objective:**

Open spina bifida is rarely observed in animals due to high levels of perinatal mortality and frequent euthanasia. To the best of our knowledge, we present the first case of spina bifida in a dog was diagnosed in‐utero and then followed post‐partum.

**Methods:**

A 3‐year‐old Poodle was presented with twin pregnancy. Radiographic and ultrasonographic findings were suggestive of vertebral malformation and open spina bifida with myelomeningocele in one foetus. Conservative treatment was given but the puppy died 3 days after birth. Thereafter, anatomical and histopathological analysis of several organs was performed to characterise the disease.

**Results:**

When the twins were born, one puppy had a linear dorsal midline cutaneous defect extending from the level of vertebrae L2–L6. R Radiographic examination showed several congenital vertebral malformations involving the thoracic segment, lumbar segment, sacrum and scapula. Histopathological examinations confirmed the presence of open spina bifida and identified additional abnormalities in several internal organs.

**Conclusions:**

This case presents a complete characterisation of open spina bifida, before birth and after death, using imaging and histopathology techniques.

## Graphical abstract

The current case reports an open spina bifida, in a Poodle puppy. Ultrasonography findings and radiographs of the dam during the third trimester identified two puppies, one with severe spina bifida. Postpartum, the affected puppy was confirmed to have undeveloped L2–L6 with paraplegia. Radiographs showed one pair of missing ribs and abnormally connected thoracic limb bones. Anatomical and histopathological analyses of several organs were performed to characterise the disease.

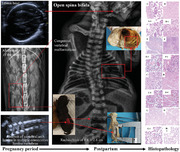

## 1 INTRODUCTION

Spina bifida is the most common congenital neural tube defect (NTD). It is caused by failed neural tube closure during early embryogenesis and results in failure of development of the vertebral arch. This may be accompanied by abnormal formation of the spinal cord and/or associated skin and subcutaneous tissue defects (Brown et al., 2020; Endalifer & Diress, 2020; Wu et al., 2021). Based on the integrity of the overlying skin and subcutaneous tissue, spina bifida malformation can be divided into open spina bifida (spina bifida aperta) and closed spina bifida (spina bifida occulta). The resulting clinical signs depend on the region of the spine affected, but usually involve pelvic limb ataxia or paralysis. The cause of spina bifida is rarely ascertained; however, toxin exposure, in‐utero stress and nutritional imbalances such as folate deficiency during pregnancy have been postulated along with genetic susceptibilities (Mitchell et al., 2004). One or several puppies in a litter may be affected.

Spina bifida and other spinal‐cutaneous malformations, such as dermoid sinus, have been widely reported in various dogs and cat breeds, including in Cane Corso (Blondel et al., 2021), Bulldog (ín Muñiz et al., 2020; Kopke et al., 2019; Motta et al., 2012; Ployart et al., 2013; Shamir et al., 2001), Dachshund (Barrios et al., 2014), Chinese Crested dog (Kiviranta et al., 2011), Swedish Vallhund (Kiviranta et al., 2011), Burmese cat (Kiviranta et al., 2011), Pekingese dog (Ruberte et al., 1995) and Yorkshire terrier (Fatone et al., 1995). However, open spina bifida is rarely observed in animals due to high levels of perinatal mortality and frequent euthanasia. While previous studies have reported spina bifida in dogs and cats, to the best of our knowledge, we present the first case of spina bifida in a dog was diagnosed in utero and then followed postpartum.

The current case reports a congenital vertebral malformation, open spina bifida, in a Poodle puppy. Ultrasonographic findings and radiographs of the dam during the third trimester identified two puppies, one with severe spina bifida. Postpartum, the affected puppy was paraplegic and had a lumbar myelomenigocele with dorsal midline skin and subcutaneous tissue defect extending from L2 to L6. Radiographs confirmed underdevelopment of the L2–L6 vertebrae and identified a pair of missing ribs and abnormally connected thoracic limb bones. The newborn died 3 days after birth and then a postmortem examination was performed.

## 2 CASE PRESENTATION

### 2.1 Pregnancy period

A 3‐year‐old Poodle dog was presented at 40−45 days of pregnancy. There had been no history of trauma or discomfort and no known exposure to teratogens. During the pregnancy, the dog was fed ‘Origen ©, TM; Six Fish’ adult dog food without folic acid supplementation.

**FIGURE 1:**
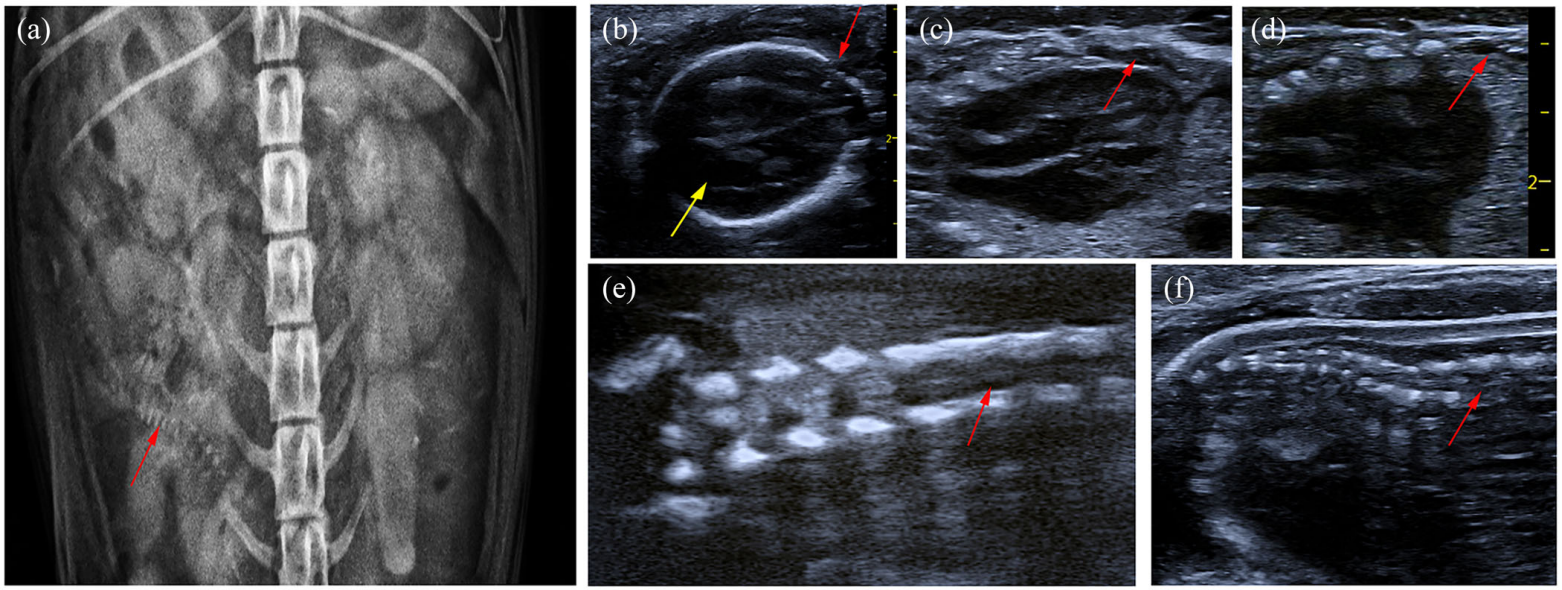
Radiographs and ultrasound images obtained during pregnancy. (A) Spinal cord abnormalities are noted in the right foetus (red arrow). (B) ‘Lemon head’ sign in the puppy, indicating cranioschysis, a typical abnormality associated to spina bifida in humans (red arrow), in addition, severe hydrocephalus is observed (yellow arrow). (C, D) No abnormalities are seen in cardiac ultrasound, but disordered thoracic spine arrangement can be seen (red arrow). (E, F) Absence of vertebral arch lamina in multiple consecutive lumbar vertebrae (rachischisis).

Prenatal radiographs (Figure 1A) showed two foetuses, one with a defect in the vertebral column and vertebral widening, suggestive of spina bifida.

Additionally, cranial ultrasonography (Figure 1B) revealed severe hydrocephalus and deformation of the frontal bone, resembling the imaging signs of human cranioschisis (‘lemon head’). Ultrasonography revealed abnormalities in thoracic spine arrangement (Figure 1C and D) and the absence of vertebral arch lamina in multiple consecutive lumbar vertebrae (Figure 1E and F).

Surgical intrauterine repair of the myelomeningocele was recommended. However, after considering the surgical risk and medical cost, the owner chose conservative treatment, which included 20 mg/kg amoxicillin‐clavulanic acid and 10 mg/kg metronidazole per os twice daily.

### 2.2 Postpartum

**FIGURE 2:**
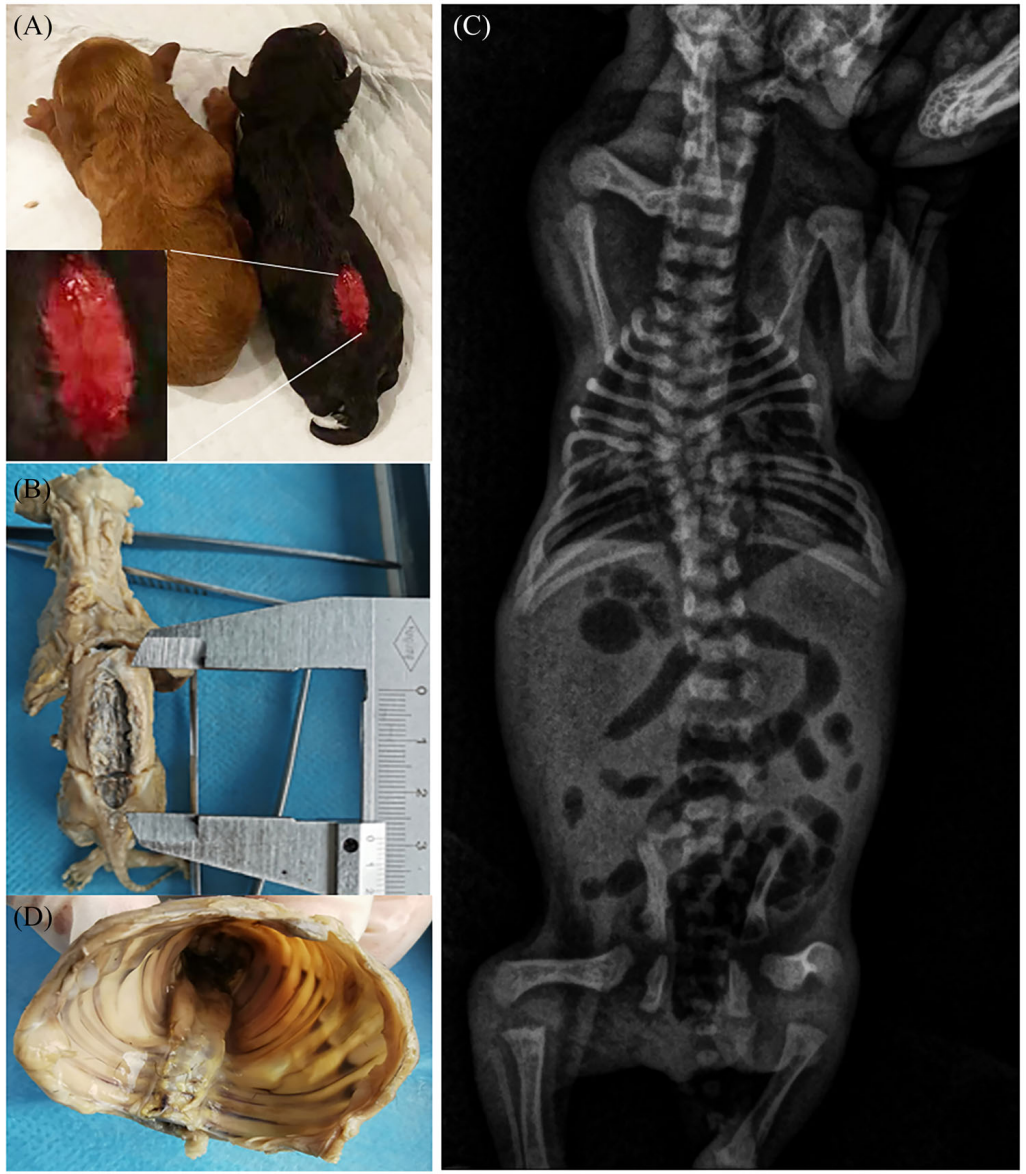
Diagnosis of open spina bifida in a neonate Poodle dog. (A) The rachischisis between L2 and L6 with skin defect. (B) The gap size is 0.8 cm × 2.6 cm. (C) Multiple malformations are seen involving the thoracic and lumbar spines, sacrum, ribs, and scapula. (D) Rib malformations are seen.

The Poodle gave birth to two male puppies: one brown and one black in colour. The brown puppy was grossly normal, but the black puppy had hydrocephalus and a myelomenigocele in the lumbar segment of the vertebral column extending from L2 to L6 (Figure 2A) of 0.8 × 2.6 cm (Figure 2B). The puppy displayed hindlimb paralysis and urinary and faecal incontinence. Spinal reflexes and deep nociception were normal, and no pain was evident on spinal palpation.

Radiography (Figure 2C) of the newborn revealed right scapular dysplasia, one pair of missing ribs, incomplete bilateral ossification of the 5th and 6th ribs, and multiple fused and ‘fanned‐out’ ribs. Multiple malformations were present in the thoracic T4–T8 vertebrae, including congenital scoliosis, butterfly vertebrae, hemivertebrae, vertebral malsegmentation defects and fused vertebrae. In the lumbar spine, malformations included L1–L2 fusion, absence of lamina between L3 and L7 vertebrae, absence of L2–L6 dorsal spinous processes and widened interpedicular distance. Sacrum dysplasia was also observed.

Antibiotics (cephalosporin, 5 mg/kg, sc, qid) and anti‐inflammatories (flunixin meglumine, 0.5 mg/kg, iv, qid) injections were administered, and the L2–L6 dorsal midline skin defect associated with the myelomenigocele was repaired using simple interrupted sutures, but the puppy died 3 days later. After obtaining informed consent from the owner, a postmortem examination was performed which confirmed the aforementioned abnormalities. During the necropsy, rib malformations and primary spinal fissure were observed (Figure 2B and D). The brain tissue was at liquefactive necrosis stage and intestinal and urinary bladder haemorrhage was observed. Other body tissues and organs showed no gross abnormalities. Tissue samples from several organs were preserved on 10% formaldehyde for histopathological analysis.

### 2.3 Histopathology

**FIGURE 3:**
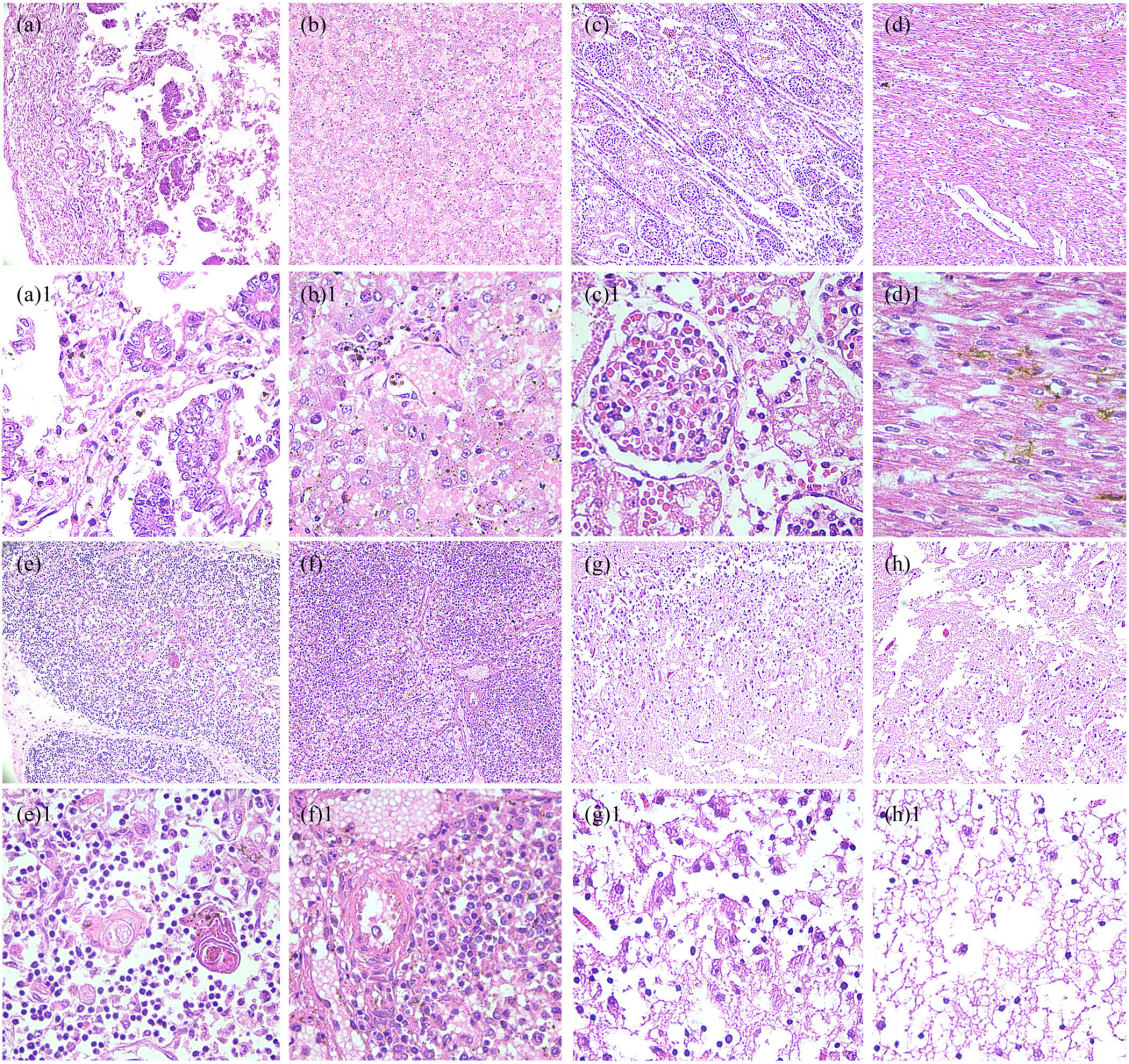
A series of pathological changes are observed in multiple organs on haematoxylin and eosin staining. **(A–H)** ×100 (scale bar = 100 μm). **(A‐1 to H‐1)** ×400 (scale bar = 400 μm). (A, A‐1) Necrotised intestinal villi and glands into the intestinal lumen; (B‐1) Diffuse hepatic cells necrosis with severe cholestasis. (B) Hepatic cells showing oedema, granular degeneration, and steatosis. (C) Disintegrated kidney glomerular structures, glomerular capillaries oedema, and diffuse congestion. (C‐1) Hydropic degeneration, swelling, vacuolisation, and reduced proximal tubule epithelial cells. (D) Atrophied and ruptured myocardial fibres with granular degeneration. (D‐1) Interstitial oedema, and heterophilic infiltration. (E, E‐1) Sparsely distributed thymic cells and lymphocytes due to thymic atrophy. (F) Decreased number of lymphocytes and increased number of splenic trabeculae. (F‐1) Splenic atrophy. (G) Brain parenchyma damage in the cerebral cortex and (H) Medulla. (G‐1, H‐1) Cellular apoptosis and necrosis in (G‐1) the cortex and (H‐1) medulla.

Evaluation of H&E stained sections (Figure 3) showed cerebral, splenic, hepatic, renal, intestinal and cardiac pathologies. Histopathology revealed necrosis and luminal shedding of the intestinal villi (Figure 3A and A‐1) and sparsely arranged smooth muscle fibres. Liver histology showed the absence of most of the hepatic cords and sinusoids as well as diffuse hepatic cell necrosis with severe cholestasis (Figure 3B‐1); the surviving hepatic cells showed oedema, granular degeneration and steatosis (Figure 3B). Renal glomerular structures were poorly defined, and there was glomerular capillary oedema and diffuse congestion (Figure 3C). Hydropic degeneration, swelling and vacuolisation were observed in the proximal tubule epithelial cells (Figure 3C‐1). Cardiac histopathology showed atrophied and ruptured myocardial fibres with granular degeneration (Figure 3D), interstitial oedema and heterophilic infiltration (Figure 3D‐1). The thymic cells and lymphocytes were sparsely distributed, as their number was reduced due to thymic atrophy (Figure 3E and E‐1). Similarly, splenic trabeculae were increased (Figure 3F), with hemosiderin deposition in the periarterial lymphatic sheath due to splenic atrophy (Figure 3F‐1). Extensive damage was observed in the brain parenchyma, including the cerebral cortex (Figure 3G) and medulla oblongata (Figure 3H), due to cell apoptosis and necrosis (Figure 3G‐1 and H‐1).

## 3 DISCUSSION

To the best of our knowledge, this is the first case report of open spina bifida diagnosed during the dam's pregnancy. Although computed tomography and magnetic resonance imaging are the methods of choice for diagnosing open spina bifida, ultrasonography is routinely used in human medicine and was chosen here considering the radiation factors and accessibility. During a third‐trimester prenatal ultrasound scan, we were able to identify several findings including cranioschysis (‘Lemon head sign’), hydrocephalus, vertebral body defects (VBDs) and absence of vertebral arch lamina in multiple consecutive lumbar vertebrae. The ability to document these key clinical features in utero facilities the possibility of early treatment of open spina bifida in dogs. Prenatal closure is the most favourable clinical procedure in human patients. This surgical intervention delays the disease progression by preventing cerebrospinal fluid leakage and infection of the myelomeningocele (Riley et al., 2019) and improves neurologic outcomes (Radic et al., 2019). Severe maternal and foetal/neonatal complications following such procedures are rare (Moehrlen et al., 2021) and foetal surgery for spina bifida should therefore be regarded as a standard therapeutic option in highly specialised centres (Dewan & Wellons, 2019). While intrauterine foetal interventions have been also described in dogs, they are not in widespread use. Intrauterine surgery was offered but declined in this patient.

The postpartum physical, radiographical and histopathological examinations confirmed the presence of open spina bifida and identified additional abnormalities beyond those that had been detected in utero. Spina bifida may be associated to other skeletal and organs deformities that affect multiple body systems including the nervous system, cardiovascular system, digestive system, urinary system and immune system. The specific abnormalities found in this puppy comprised almost identical characteristics to those described in the human form (Rhode, 2021), and were severe in nature. It is widely acknowledged that spina bifida can lead to significant damage to the nervous system (Rocque et al., 2021), and this was confirmed by histopathology. Additionally, we observed shrinkage of thymus and spleen. Thymus as the central immune organ is most active during foetal and postnatal development, and its degeneration is likely to take place after adulthood. Consequently, the atrophy of the central immune organ of this foetus is an indication of serious immune system abnormality. We hypothesise that the maldevelopment of neural tube to closure during embryonic development, which is the root cause of spinal bifida, may have an adverse impact on the development of the immune system and other organ systems (Liptak et al., 2013). Haemorrhage, necrosis and other pathologies of the liver and kidney may have been a result of secondary processes associated with foetal decline and aberrant metabolism. Faced with such a severe disease, prevention is the main priority, and nutrition is a key determinant of neural tube defects. In particular, there is a strong link with folic acid (Omer et al., 2014) and in humans, preventative methods for these serious birth defects include adequate folic acid intake. This case highlights the necessity for folic acid supplementation in animals and also confirms the importance of ultrasound examination during pregnancy.

## 4 CONCLUSION

This report characterises a case of spina bifida in a Poodle puppy pre‐ and postnatally. It describes an uncommon spinal developmental condition associated with other organ malformations in dogs and provides a starting point for further research in this field.

## AUTHOR CONTRIBUTIONS

Yue Chen: Conceptualisation; data curation; writing—original draft; writing—review & editing. Dan Su: Conceptualisation; writing—original draft; writing—review & editing. Xiaorong Sun: Conceptualisation; writing—review & editing. Wenjuan Gui: Data curation.

## CONFLICT OF INTEREST STATEMENT

The authors declare that they have no known competing financial interests or personal relationships that could have appeared to influence the work reported in this paper.

## FUNDING

This research was supported by Sichuan Science and Technology Program (2021JDRC0061).

## ETHICS STATEMENT

The authors confirm that the ethical policies of the journal, as noted on the journal's author guidelines page, have been adhered to. No ethical approval was required as this is a case report article with no original research data.

### PEER REVIEW

The peer review history for this article is available at https://publons.com/publon/10.1002/vms3.1266.

## Data Availability

Data openly available in a public repository that issues datasets with DOIs.
